# Time‐Lapse Culture Improves Development and Pregnancy Outcomes of Sibling Normally Fertilized Oocytes: A Prospective Multicenter Study

**DOI:** 10.1002/rmb2.70077

**Published:** 2026-07-24

**Authors:** Yasuyuki Mio, Takeshi Kuramoto, Takafumi Utsunomiya, Seung Chik Jwa, Keitaro Yumoto, Panagiota Tsounapi

**Affiliations:** ^1^ Reproductive Centre, Mio Fertility Clinic Tottori Japan; ^2^ Kuramoto Women's Clinic Fukuoka Japan; ^3^ St. Luke Clinic Oita City Oita Japan; ^4^ Department of Obstetrics and Gynecology Jichi Medical University Tochigi Japan

**Keywords:** ART, benchtop incubator, clinical pregnancy, embryo culture, embryo development, time‐lapse incubator

## Abstract

**Purpose:**

To evaluate whether time‐lapse monitoring improves embryological outcomes and clinical pregnancy rates compared to conventional culture in a sibling‐oocyte study.

**Methods:**

This multicenter‐study involved 205 first‐time retrieval cycles (*n* = 3244 mature oocytes). Sibling‐oocytes from patients with ≥ 4 oocytes were randomized into conventional and time‐lapse groups. The primary outcome was the good‐quality blastocyst rate. Key secondary outcomes included Day‐2 Good Quality Embryo (GQE) rates and clinical pregnancy rates for both fresh and frozen–thawed embryo transfers (FET).

**Results:**

The time‐lapse group exhibited a significantly higher GQE rate on Day 2 (42.1% vs. 35.3%, *p* < 0.01) and a higher good‐quality blastocyst rate (44.1% vs. 38.1%, *p* < 0.01) compared to the conventional group. While clinical pregnancy rates showed a higher trend in the time‐lapse group for both fresh ET (44.4% vs. 15.8%) and FET (45.3% vs. 36.7%), these differences did not reach statistical significance. Time‐lapse monitoring successfully identified embryos with early pronuclear disappearance as early as 14 h post‐insemination. Morphokinetic analysis revealed that abnormal cleavage patterns were associated with significantly lower blastocyst development potential compared to normal cleavage (*p* < 0.01).

**Conclusion:**

Time‐lapse culture provides a stable environment that enhances embryo quality. While clinical pregnancy rates showed a positive trend, further large‐scale studies are needed to confirm the impact on live birth rates.

## 1 Introduction

In assisted reproductive technology (ART), the non‐invasive selection of embryos with high implantation potential would greatly improve treatment efficiency. The development of in vitro culture systems that closely replicate the in vivo environment is a major challenge that the field of assisted reproduction is constantly facing. In conventional benchtop incubators, culture the embryos must be removed for observation at least once daily, yielding only intermittent and fragmented information, while the continuous monitoring of the developmental processes is not possible.

The introduction of incubators equipped with time‐lapse imaging systems has addressed this limitation by enabling continuous observation and recording of embryo development [1, 2]. This technology allows assessment of developmental events that previously were inaccessible, such as (a) the second polar body extrusion, (b) the pronuclear formation and number, (c) syngamy, (d) the first to third cleavage divisions, (e) onset of compaction, and (f) timing of blastocoel formation. As a result, detailed embryonic developmental timelines have been established [3, 4, 5, 6].

Several studies have reported that time‐lapse culture improves developmental outcomes compared with conventional culture. Reported advantages include improved cleavage dynamics and higher blastocyst formation rates [7, 8, 9, 10, 11]. Others have emphasized the predictive value of early cleavage patterns; particularly the presence or absence of direct cleavage (DC) and reverse cleavage (RC), for selecting embryos with higher implantation potential at the cleavage stage [12, 13, 14, 15]. These findings suggest that time‐lapse culture may be especially useful for the evaluation of the early embryonic development.

However, evidence remains conflicting. Some studies have found no significant improvement in culture outcomes with time‐lapse monitoring [16, 17, 18]. Furthermore, embryos exhibiting DC or RC that nevertheless reach the blastocyst stage may display comparable euploidy rates and implantation potential to normally cleaving embryos [19, 20, 21, 22]. These findings suggest that many zygotes with abnormal chromosomal segregation fail to develop to the blastocyst stage, whereas blastocysts derived from abnormal cleavage embryos may still predominantly consist of euploid blastomeres. Conversely, other reports indicate that time‐lapse culture not only increases blastocyst formation rates but also enhances the yield of euploid embryos [23, 24].

More recently, the integration of artificial intelligence (AI) algorithms with time‐lapse monitoring has gained increasing attention. Several studies have demonstrated that AI‐based embryo evaluation can improve the selection of embryos with higher implantation potential [25, 26, 27, 28, 29, 30, 31, 32], and correlations between AI scores and preimplantation genetic testing results suggest the possibility of non‐invasive identification of euploid embryos [33, 34, 35].

Taken together, the clinical value of time‐lapse culture compared with conventional culture remains unresolved, and considerable debate persists. To address this gap, we conducted a multicenter study to clarify the clinical utility of time‐lapse culture. To minimize bias, we restricted inclusion to women undergoing their first oocyte retrieval and utilized a prospective sibling‐oocyte allocation design. This approach allowed us to randomly allocate sibling oocytes from the same patient to either time‐lapse or conventional culture, ensuring a direct and controlled comparison of embryological and clinical outcomes.

## 2 Materials and Method

### 2.1 Study Design

We designed a prospective sibling‐oocyte allocation study to comparatively analyze the culture outcomes between two distinct incubation systems:

1. Conventional, humidified benchtop incubators (5% O_2_, 6% CO_2_, and 89% N_2_); conventional group
2. Time‐lapse monitoring incubators (non‐humidified and humidified; 5% O_2_, 6% CO_2_, and 89% N_2_); time‐lapse group.

This study was conducted across three affiliated facilities of JISART (the Japanese Institution for Standardizing Assisted Reproductive Technology). Patients who provided written informed consent were enrolled, and comparative developmental data were prospectively obtained from both the time‐lapse and conventional incubator cohorts. To acknowledge platform heterogeneity, the time‐lapse cohort included both humidified (Geri) and non‐humidified (EmbryoScope+) systems already in clinical use.

### 2.2 Participants

Between April 1, 2022, and December 31, 2023, 586 patients across the three JISART‐affiliated facilities who were scheduled for their first oocyte retrieval were provided with a written explanation of the study. Consent for participation was obtained from 389 cases.

The final analysis cohort was restricted to 205 cycles (205 cases) that met the following inclusion criteria:

- Patients provided written informed consent prior to the commencement of treatment.
- The patient was undergoing IVF, ICSI, or IVF/ICSI with autologous oocytes during their first oocyte retrieval.
- Patients with ≥ 4 mature oocytes retrieved and available for sibling‐oocyte allocation.

During the study period (April 1, 2022, to December 31, 2023), data were collected from 205 cycles that fulfilled the above‐mentioned criteria. The choice of controlled ovarian stimulation (COS) protocol, oocyte retrieval method, insemination/fertilization method, endometrial preparation, luteal phase support, and other IVF laboratory procedures were left to the discretion of each participating clinic, adhering to their respective established standard‐of‐care protocols.

**FIGURE 1:**
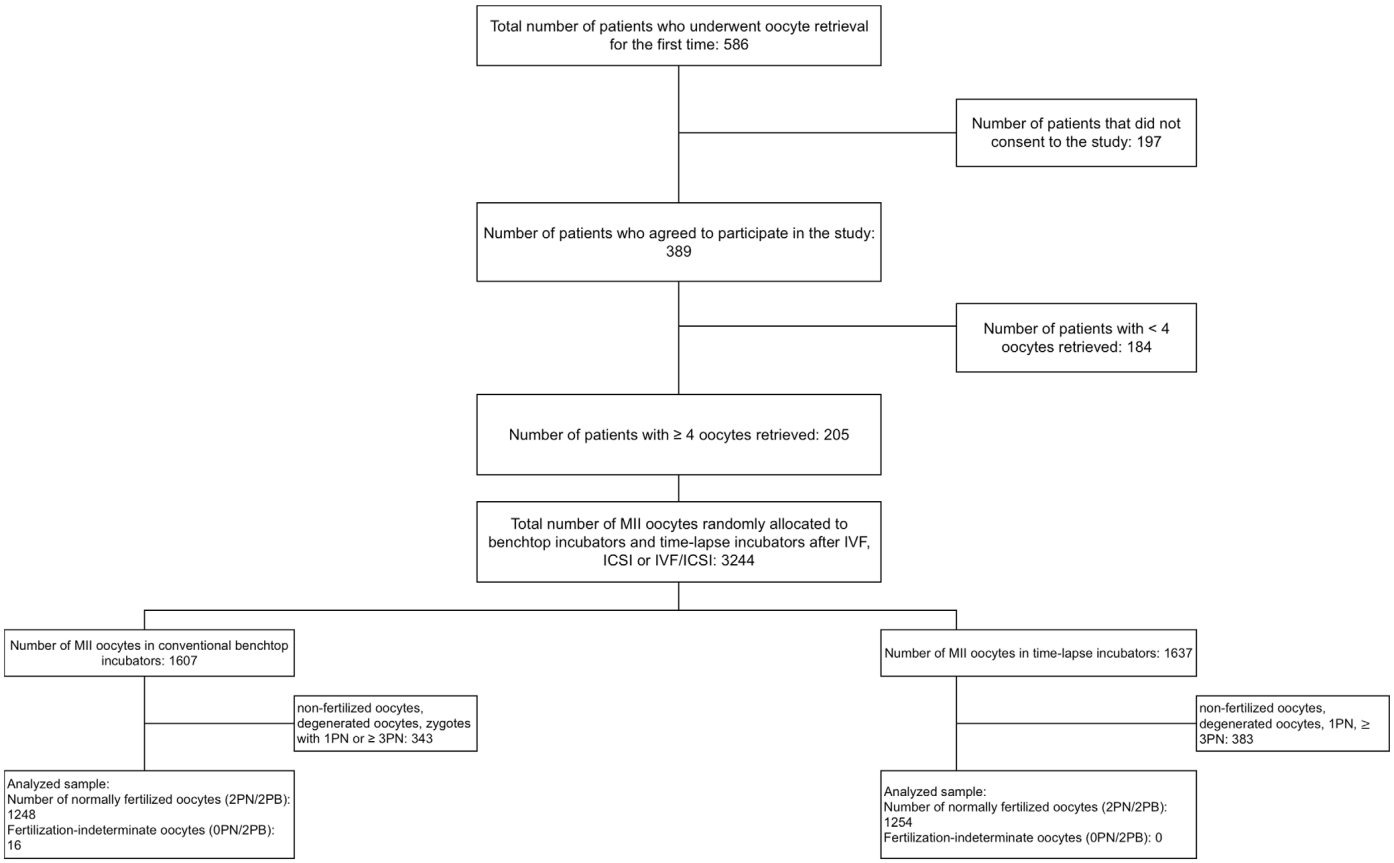
CONSORT flow diagram of patient recruitment and sibling‐oocyte allocation.

In the initial oocyte retrieval of the included patients, a total of 3244 oocytes underwent IVF, ICSI, or IVF/ICSI. These oocytes/zygotes were subsequently randomized into two groups and cultured separately: the conventional group (*n* = 1607) and the time‐lapse group (*n* = 1637) (Figure 1).

The conventional incubators used in this study were the benchtop type incubators routinely employed by each clinic. The time‐lapse incubators used were either non‐humidified or humidified time‐lapse incubators (Embryoscope^+^, Geri) already available and in use at each facility. Additionally, culture media were used according to each clinic's standard protocol and a mineral oil overlay was used to prevent evaporation.

### 2.3 Ethical Approval

Approval for publication of the study was applied for and granted by the Ethics Committee of the JISART (2021‐06). The study does not contain any patient‐identifying information.

### 2.4 Sperm Preparation

Sperm samples were purified and adjusted to ≤ 10 × 10^6^ spermatozoa/mL by the combined method of centrifugation on density gradients (Percoll; 40%, 70%, and 90%; 1500 rpm, 30 min) and swim‐up.

### 2.5 Oocyte Allocation and Experimental Design

Sibling oocytes from each patient were distributed between the conventional benchtop and time‐lapse incubation groups in a 1:1 ratio using an alternating allocation protocol with a concealed/randomized starting arm. To eliminate selection bias, for each patient cohort, the designation of the first oocyte (Position 1) to either the time‐lapse or conventional system was determined by a pre‐specified schedule managed independently of the performing embryologist. This allocation was completed during the denudation process and prior to any morphological assessment, whereupon subsequent sibling oocytes were alternately assigned.

The allocation timing was tailored to the fertilization method:

- *Intracytoplasmic Sperm Injection (ICSI)*: Following denudation and the exclusion of immature oocytes (MI/GV), mature MII oocytes were assigned to their respective systems immediately prior to injection based on the concealed sequence
- *Conventional IVF (c‐IVF)*: Sibling oocytes were inseminated in 4‐well dishes and maintained in a shared conventional incubator during the co‐incubation period to ensure identical fertilization conditions. Post‐denudation, oocytes were allocated to their pre‐assigned incubation systems.

### 2.6 Assessment of Fertilization Status

On Day 1, all participating centers performed formal fertilization assessment for every single oocyte that was included in the study according to their standard operating procedures, avoiding omissions in fertilization verification reported in some time‐lapse systems [36]. Fertilization was assessed at 17 ± 1 h post‐insemination. Oocytes demonstrating 2PN were defined as normally fertilized. In cases where pronuclei were not observed during the fertilization check but subsequent cleavage occurred, the embryos were classified as “fertilization undetermined” and remained in culture.

### 2.7 Continuous Culture of the 2PN Zygotes, Embryo Transfers (ETs) and Cryopreservation

In both groups, embryos were cultured in one‐step medium under a mineral oil overlay (heavy oil for time‐lapse group and washed oil for conventional group). This continuous culture medium was designed to support development from the zygote to the blastocyst stage without renewal. By utilizing a one‐step system, we ensured that embryos in the time‐lapse group remained completely undisturbed throughout the entire culture period, whereas embryos in the conventional group were only removed briefly for scheduled morphological assessments.

The decision to perform a fresh embryo transfer (ET) or a “freeze‐all” cycle was determined by clinical criteria—including progesterone levels, endometrial thickness, and OHSS risk—independent of the study group allocation. To ensure an unbiased comparison within this sibling‐oocyte design, the embryo with the highest morphological grade (based on Gardner's criteria or institutional GQE standards) was prioritized for transfer. Crucially, both clinicians and patients remained blinded to the incubator assignment during the selection and transfer process. In cases where embryos from both groups shared identical morphological grades, selection followed a standardized laboratory workflow, which contributed to the observed distribution of fresh ETs. Fresh ET at either the cleavage or blastocyst stage was permitted according to the criteria of each facility. For both groups, cleavage‐stage fresh ET was restricted to good‐quality embryos (GQE), defined as ≥ 4‐cell embryos with less than 20% fragmentation at 40 h post‐insemination. Embryo cryopreservation was performed at either the cleavage or blastocyst stage based on institutional grading standards using the vitrification method.

### 2.8 Morphological Evaluation of Embryos

Early‐stage embryos were assessed using modified Veeck's criteria in accordance with the Istanbul Consensus [36, 37]. 2PN zygotes were classified into three categories based on cytoplasmic fragmentation and blastomere size:

- *Category 1*: ≤ 20% fragmentation (Grade 1).
- *Category 2*: > 20% to < 40% fragmentation (Grade 2).
- *Category 3*: ≥ 40% fragmentation (Grade 3).

Initiation of a blastocyst development was considered when a blastocoel was visible. Morphologically good‐quality blastocysts were defined as those graded ≥ 4BB according to Gardner's criteria [38, 39]. Embryo evaluations were performed independently by at least two senior embryologists.

### 2.9 Statistical Analysis

Baseline clinical characteristics, including maternal age, BMI, and infertility duration, are presented as mean ± standard deviation (SD). Since a sibling‐oocyte design was employed, these patient‐specific parameters were identical for both study arms and were not subject to comparative statistical testing.

Differences in categorical embryological outcomes—including fertilization rates, GQE rates, and blastocyst development rates—were analyzed using the Chi‐square test. To account for the clustering of sibling oocytes within the same patient cycle and adjust for intra‐patient correlation, a generalized estimating equation (GEE) was utilized. For the analysis of morphokinetic parameters within the time‐lapse group, the normal cleavage group (1 → 2 cells) served as the reference for comparisons against the abnormal cleavage patterns (1 → 2 → 3 and 1 → 3).

To evaluate the clinical impact of the incubation system on FET outcomes, univariate and multivariate logistic regression analyses were performed to calculate crude and adjusted odds ratios (ORs) with 95% confidence intervals (CIs). The multivariate model adjusted for maternal age, BMI, smoking status, and primary infertility diagnosis. Furthermore, to account for the potential correlation between multiple FET cycles performed for the same patient (*n* = 361 cycles from 156 patients), the GEE model was extended to the clinical analysis, utilizing a logit link function and an exchangeable correlation structure to account for within‐patient correlation across repeated transfers. Statistical significance was defined as *p* < 0.05.

The good‐quality blastocyst rate was pre‐specified as the primary endpoint. Sample size was calculated based on a predicted 5% increase in the good‐quality blastocyst rate from a baseline of 38% (conventional group). To achieve 80% power with a significance level of α = 0.05, a minimum of 950 embryos per group was required. The final analysis included 2063 embryos (998 Conventional vs. 1065 time‐lapse), ensuring the study was adequately powered for the primary outcome. Clinical pregnancy outcomes were considered exploratory secondary endpoints.

## 3 Results

In this multicenter study, we analyzed 205 initial oocyte retrieval cycles from three participating institutions. Inclusion was restricted to patients who provided informed consent and yielded ≥ 4 mature oocytes for sibling‐oocyte allocation (Figure 1). Patient demographics and baseline clinical characteristics are summarized in Table 1. The mean ages for female and male partners were 33.2 ± 4.3 and 34.9 ± 5.6 years, respectively. Female participants had a mean serum anti‐Müllerian hormone (AMH) level of 5.64 ± 4.21 ng/mL, a mean body weight of 53.0 ± 8.5 kg, and a mean body mass index (BMI) of 20.9 ± 3.1 kg/m^2^. The mean duration of infertility prior to the first oocyte retrieval was 27.3 ± 22.1 months. Across the 205 study cycles, a total of 3907 oocytes were retrieved (mean 19.1 ± 9.6 per cycle), of which 3244 were confirmed as mature (MII stage; mean 15.8 ± 8.8 per cycle). Following fertilization via IVF or ICSI, fertilization status was formally assessed on Day 1.

**TABLE 1:** Baseline demographic and clinical characteristics of the study population.^a^

	*n* (%) or Mean ± SD	Median (Range)
Female age (y.o.)	33.2 ± 4.3	33 (25–42)
Male partner's age (y.o.)	34.9 ± 5.6	34 (24–51)
Number of previous pregnancies	0.7 ± 1.1	0 (0–7)
Number of previous live births	0.3 ± 0.6	0 (0–4)
Duration of infertility (months)	27.3 ± 22.1	24 (1–176)
Current smoker	7 (3.4%)	—
BMI (kg/m^2^)	20.9 ± 3.1	20.3 (15.7–38.2)
Infertility diagnosis		
Tubal factor	13 (6.3%)	—
Male factor	56 (27.3%)	—
Endometriosis	26 (12.7%)	—
PCOS/ovulation disorders	43 (21.0%)	—
Uterine factor^b^	7 (3.4%)	—
Unexplained	92 (44.9%)	—
AMH (ng/ml)	5.64 ± 4.21	4.36 (0.5–25.6)
Number of retrieved oocytes	19.1 ± 9.6	18 (4–51)
Number of mature oocytes	15.8 ± 8.8	14 (4–48)
Number of freeze all cycles	177 (86.3%)	—
Fertilization method		
IVF	45 (22.0%)	—
ICSI	73 (35.6%)	—
IVF/ICSI	87 (42.4%)	—

Abbreviations: AMH, anti‐Müllerian hormone; BMI, body mass index; ICSI, intracytoplasmic sperm injection; IVF, in vitro fertilization; PCOS, polycystic ovary syndrome; SD, standard deviation.

^a^ All participants underwent oocyte retrieval for the first time.

^b^ Includes uterine fibroids and endometrial polyps.

Following sibling‐oocyte allocation, 1607 MII oocytes were distributed to the conventional group and 1637 to the time‐lapse group. Normal fertilization (2PN/2 PB) rates were comparable between the two cohorts at 77.7% (*n* = 1248) and 76.6% (*n* = 1254), respectively. By Day 2 of development, the rate of good‐quality embryos (GQE) was significantly higher in the time‐lapse group compared to the conventional group (42.1% vs. 35.3%; *p* < 0.01). In contrast, no significant difference was observed in the 4‐cell stage rate between the two groups (55.6% vs. 55.2%; *p* > 0.05).

Extended culture was performed for 998 embryos in the conventional group and 1065 in the time‐lapse group. While overall blastocyst formation rates were comparable between the two groups (66.7% vs. 68.2%, respectively; *p* > 0.05), the rate of good‐quality blastocysts was significantly higher in the time‐lapse cohort (44.1% vs. 38.1%; *p* < 0.01). This suggests that the stable environment provided by the time‐lapse system primarily enhances the morphological quality of the developing blastocysts rather than the total developmental rate. While baseline good‐quality embryo (GQE) rates varied slightly between participating centers, no significant interaction was found between clinic site and the primary study outcomes.

Notably, the use of time‐lapse monitoring eliminated the occurrence of unclassifiable fertilization, whereas 1.0% (*n* = 16) of embryos in the conventional group were categorized as “indeterminate fertilization” embryos due to the lack of visible pronuclei at the time of observation. Although Day 2 GQE rates for these unclassified embryos were comparable to those of 2PN embryos, their 4‐cell development rate was lower. Subsequent extended culture of these embryos (*n* = 15) achieved a blastocyst formation rate of 66.7% (*n* = 10) and a good‐quality blastocyst rate of 46.7% (*n* = 7), which was comparable to the developmental potential of normally fertilized embryos (Table 2).

**TABLE 2:** Embryological and clinical outcomes of sibling embryos: Conventional vs. time‐lapse incubation in fresh cycles.

	Culture with conventional incubators^a^	Culture with time‐lapse incubators	*p* ^b^
No of MII oocytes (*n*)	1607	1637	
Normally fertilized oocytes (2PN/2PB)	1248	1254	
2PN/2PB (%)	77.7% (1248/1607)	76.6% (1254/1637)	0.47
Day‐2 GQE (%)	35.3% (440/1248)	42.1% (528/1254)	**< 0.01**
Day‐2 4‐cell embryos (%)	55.2% (689/1248)	55.7% (698/1254)	0.82
No of extended cultures (*n*)	998	1065	
Embryos that became blastocysts (%)	66.7% (666/998)	68.2% (726/1065)	0.49
Good‐quality blastocysts (%)	38.1% (380/998)	44.1% (470/1065)	**< 0.01**
Embryo utilization rate (%)	50.3% (628/1248)	52.2% (655/1254)	0.34
No of fresh ET (*n*)	19	9	
Clinical pregnancy rate after fresh ET (%)	15.8% (3/19)	44.4% (4/9)	0.16
Miscarriage rate (%)	0.0% (0/3)	50.0% (2/4)	0.43
Fertilization‐indeterminate oocytes (0PN/2PB)	16	0	
0PN/2PB (%)	1.0% (16/1607)	—	—
Day‐2 GQE (%)	31.3% (5/16)	—	—
Day‐2 4‐cell embryos (%)	37.5% (6/16)	—	—
No of extended cultures	15	—	—
Embryos that became blastocysts (%)	66.7% (10/15)	—	—
Good‐quality blastocysts (%)	46.7% (7/15)	—	—

*Note:* The good‐quality blastocyst rate was the pre‐specified primary outcome. Clinical pregnancy rates were analyzed as exploratory secondary endpoints.

Abbreviations: ET, embryo transfer; GQE, good‐quality embryo; PB, polar body; PN, pronucleus.

^a^ Fertilization confirmation in benchtop incubators was done within 17 h after insemination.

^b^ *p*‐values are assessed with a chi‐square test. Statistical significance appears in bold.

Fresh embryo transfer (fresh ET) using morphologically good‐quality embryos was performed in 19 cycles for conventional culture and 9 cycles for time‐lapse culture. Clinical pregnancy rates were 15.8% (*n* = 3) and 44.4% (*n* = 4), respectively, showing a higher tendency toward improved pregnancy rates in the time‐lapse group, although the difference was not statistically significant. Miscarriage rates were 0.0% (*n* = 0) for conventional culture and 50.0% (*n* = 2) for time‐lapse culture, with no significant difference (Table 3).

**TABLE 3:** Comparison of FET clinical outcomes for embryos derived from sibling oocytes in conventional versus time‐lapse incubators.

	Culture with conventional incubators (*n* = 158)	Culture with time‐lapse incubators (*n* = 203)	*p* ^a^
Endometrial preparation protocol			
CC + hMG	17.1% (27/158)	16.3% (33/203)	0.83
HRT	82.9% (131/158)	83.7% (170/203)	
Fertilization method			
IVF	44.9% (71/158)	44.8% (91/203)	0.98
ICSI	55.1% (87/158)	55.2% (112/203)	
Single ET	98.7% (156/158)	95.1% (193/203)	0.74
Clinical pregnancy rate per ET	36.7% (58/158)	45.3% (92/203)	0.1
Miscarriage rate	20.6% (12/58)	28.3% (26/92)	0.3
Cleavage stage ETs	*n* = 82	*n* = 93	
ET using good‐quality embryo	97.6% (80/82)	97.8% (91/93)	0.9
Clinical pregnancy rate per ET	37.8% (31/82)	40.8% (38/93)	0.68
Miscarriage rate	22.6% (7/31)	13.2% (5/38)	0.3
Blastocyst ETs	*n* = 76	*n* = 110	
ET using good‐quality embryo	97.4% (74/76)	96.4% (106/110)	0.7
Clinical pregnancy rate per ET	35.5% (27/76)	49.1% (54/110)	0.07
Miscarriage rate	18.5% (5/27)	38.9% (21/54)	0.06

Abbreviations: CC, clomiphene citrate; ET, embryo transfer; FET, frozen–thawed embryo transfer; HMG, human menopausal gonadotropin; HRT, hormone replacement therapy; IVF, in vitro fertilization; ICSI, intracytoplasmic sperm injection.

^a^ *p*‐values are assessed with chi‐square test.

Frozen–thawed embryo transfer (FET) was performed in 361 cycles among 156 patients with available frozen embryos. Clinical characteristics of FET cycles are shown in Table 4, with no differences observed in endometrial preparation or fertilization methods. Morphologically good‐quality embryos from conventional and time‐lapse cultures were transferred in 158 and 203 FET cycles, respectively. Clinical pregnancy rates were 36.7% (*n* = 58) and 45.3% (*n* = 92), showing a non‐significant trend toward higher pregnancy rates in the time‐lapse group (Table 5). Analyses by stage of transfer (cleavage‐stage vs. blastocyst‐stage) also demonstrated a trend toward higher clinical pregnancy rates with time‐lapse culture, with no significant differences in miscarriage rates.

**TABLE 4:** Univariable and multivariable logistic regression analysis for clinical pregnancy outcomes in FET cycles utilizing Generalized Estimating Equations (GEE) to account for multiple cycles per patient.

	Crude OR (95% CI)	Adjusted OR (95% CI)^a^, ^b^
Overall (*n* = 361)		
Benchtop incubators	Reference	Reference
Time‐lapse incubators	1.43 (0.93–2.19)	1.44 (0.92–2.24)
Cleavage stage ETs (*n* = 175)		
Benchtop incubators	Reference	Reference
Time‐lapse incubators	1.14 (0.62–2.09)	0.92 (0.47–1.81)
Blastocyst ETs (*n* = 186)		
Benchtop incubators	Reference	Reference
Time‐lapse incubators	1.75 (0.96–3.19)	**1.99 (1.02**–**3.87)**

*Note:* Statistical significance (*p* < 0.05) is shown in bold.

Abbreviations: CI, confidence interval; FET, frozen–thawed embryo transfer; OR, odds ratio.

^a^ Adjusted for age, BMI, smoking status and infertility diagnosis.

^b^ Results estimated using a Generalized Estimating Equation (GEE) model to account for intra‐patient correlation among multiple FET cycles.

**TABLE 5:** Blastocyst development and clinical FET outcomes stratified by the nature of the first cleavage in time‐lapse culture.

	Normal first cleavage	Direct cleavage		*p* ^a^
		Abnormal first cleavage type 1	Abnormal first cleavage type 2	
	(1 → 2 cells)	(1 → 2 → ≧ 3 cells)	(1 → ≧ 3 cells)	
Frequency of occurrence	81.5% (1022/1254)	11.0% (138/1254)	7.5% (94/1254)	
No of extended cultures	*n* = 833	*n* = 138	*n* = 94	
Embryos that became blastocysts (%)	76.1% (634/833)	55.8% (77/138)	21.3% (20/94)	**< 0.01**
Good‐quality blastocysts (%)	51.5% (429/833)	28.3% (39/138)	2.1% (2/94)	**< 0.01**
No of FET embryos^**b**^	*n* = 215	*n* = 2	*n* = 0	
Clinical pregnancy rate (%)	42.8% (92/215)	0% (0/2)	—	
Miscarriage rate (%)	28.3% (26/92)	—	—	

Abbreviation: FET, Frozen–thawed embryo transfer.

^a^ *p*‐values are assessed with chi‐square test; statistical significance (*p* < 0.05) is shown in bold.

^b^ Pregnancy and miscarriage rates in this table are calculated using the number of transferred embryos as the denominator for stratification purposes. Because these clinical endpoints are fundamentally transfer‐level outcomes, these figures should be interpreted with caution.

The clinical pregnancy outcomes for FET cycles (*n* = 361) were further analyzed using crude and adjusted odds ratios (OR) to evaluate the impact of the incubation system (Table 4). In the overall FET cohort, the use of time‐lapse monitoring was not significantly associated with an increase in clinical pregnancy in either the crude analysis (OR 1.43, 95% CI: 0.93–2.19) or the adjusted analysis (adjusted OR 1.44, 95% CI: 0.92–2.24). Similarly, for cleavage‐stage transfers (*n* = 175), no significant association was observed. However, a significant finding emerged within the blastocyst transfer subgroup (*n* = 186). While the crude analysis did not reach statistical significance (OR 1.75, 95% CI: 0.96–3.19), the association became significant after adjusting for confounding factors, including maternal age, BMI, smoking status, and infertility diagnosis (adjusted OR 1.99, 95% CI: 1.02–3.87). This indicates that when transferred at the blastocyst stage, embryos cultured in time‐lapse systems were approximately twice as likely to result in a clinical pregnancy compared to those from benchtop incubators.

Time‐lapse culture enabled detailed analysis of the first cleavage event. Among normally fertilized embryos (*n* = 1254), three groups were identified: normal cleavage from 1 → 2 cells (*n* = 1022; 81.5%), abnormal cleavage from 1 → 2 → 3 cells within 5 h (*n* = 138; 11.0%), and abnormal direct cleavage from 1 → ≥ 3 cells (*n* = 94; 7.5%) (Table 5). Blastocyst formation rates and good‐quality blastocyst rates were significantly lower in the 1 → 2 → 3 cells and 1 → 3 cells groups compared with the 1 → 2 cells group (*p* < 0.01). Furthermore, the 1 → 3 cells group had significantly lower rates than the 1 → 2 → 3 cells group for both outcomes (*p* < 0.01).

## 4 Discussion

In the present study, time‐lapse embryo culture was associated with a notable trend toward higher clinical pregnancy rates per embryo transfer compared with conventional culture. Additionally, Day 2 GQE rates and good‐quality blastocyst formation rates were significantly improved in the time‐lapse group. These results suggest that the stability of the culture environment is critical. However, it is crucial to emphasize that this study was statistically powered specifically for embryo‐level developmental endpoints, designating the good‐quality blastocyst rate as our single primary endpoint. Consequently, all clinical pregnancy analyses must be interpreted strictly as secondary or exploratory observations. This framing is particularly essential given the limited fresh ET sample size, the inherent differences between embryo‐level and patient‐level units of analysis, and the unique clinical characteristics across repeated transfer cycles. This cautious approach prevents the overinterpretation of our clinical findings and remains tightly aligned with the underlying statistical design of the study. Indeed, while conventional culture requires the daily removal of embryos for observation—introducing temperature and gas fluctuations—time‐lapse systems allow for continuous monitoring without environmental disruption.

Our findings regarding indeterminate fertilization (1.0% in the conventional group) highlight the diagnostic advantage of time‐lapse monitoring. Despite a slower initial cleavage tendency, these embryos achieved blastocyst rates comparable to 2PN embryos, suggesting that continuous monitoring is essential to salvage embryos that would otherwise be discarded or deprioritized due to missed PN visibility. These findings suggest that such cases likely represent rapid PN fading before manual observation; however, without continuous monitoring, they cannot be distinguished from abnormal fertilization (1PN or ≥ 3PN). Consequently, even if these embryos reach the blastocyst stage, they are typically prioritized lower and require specific patient consent before transfer. By providing a complete developmental record, time‐lapse monitoring allows for the confident clinical use of these ‘salvaged’ embryos, which might otherwise be excluded from the transfer pool.

The timing of embryo entry into the time‐lapse incubator appears critical and it is a practical takeaway from our data. Since the earliest PN disappearance in this study occurred at 14 h post‐insemination, we may suggest in order to maximize system performance that embryos should be placed in the system within 13 h post‐insemination to ensure no fertilization events are missed. This underscores the importance of optimizing co‐culture duration to ensure the capture of normal fertilization events while minimizing the misclassification of oocytes as unfertilized or abnormal.

The first cleavage represents a particularly dynamic developmental stage. We categorized abnormal cleavage into two patterns: (1) embryos cleaving to ≥ 3 cells within 5 h of the first division (1 → 2 → ≥ 3), and (2) embryos directly cleaving to ≥ 3 cells (1 → ≥ 3). Their frequencies were 11.0% (*n* = 138) and 7.5% (*n* = 94), respectively. Both groups showed significantly reduced blastocyst formation (1 → 2 → ≥ 3: 55.8%; 1 → ≥ 3: 21.3%) and good‐quality blastocyst rates (1 → 2 → ≥ 3: 28.3%; 1 → ≥ 3: 2.1%; *p* < 0.01) compared with normally cleaving embryos. These patterns may reflect chromosomal abnormalities: sequential rapid cleavage may involve abnormal duplication in a single blastomere, whereas direct cleavage may indicate widespread chromosomal misdistribution. However, attributing these abnormal cleavage behaviors primarily to genomic errors may be restrictive. Recent live‐imaging studies demonstrate that aberrant cell behaviors during early human cleavage can stem directly from cleavage furrow misregulation and broader cytokinetic abnormalities independent of chromosomal status [40]. Furthermore, dysfunction in cell‐cycle checkpoint regulatory mechanisms—frequently observed in developmentally compromised or postovulatory‐aged oocytes—can trigger fragmentation and abnormal cytokinesis without overt chromosomal abnormalities [41]. Regardless, these embryos are less suitable for early‐stage embryo transfer and warrant extended culture to assess their full developmental potential. Our results reinforce the value of time‐lapse morphokinetics in selecting against embryos with poor developmental potential, particularly when morphology alone on Day 2 or 3 might appear misleadingly normal.

Advances in AI‐assisted evaluation further enhance these assessments. Reports indicate that embryos ranked highest by AI can achieve clinical pregnancy rates exceeding 70% in FET cycles, surpassing traditional subjective evaluation [32]. Despite the initial investment and ongoing costs, time‐lapse culture provides non‐invasive, continuous data that may complement or reduce reliance on invasive PGT‐A, where clinical pregnancy rates for euploid embryos range from 60% to 70% [42]. The significant improvement in pregnancy odds for blastocyst FET (Adjusted OR 1.99; Table 4) after utilizing a GEE model to account for intra‐patient correlation suggests that the superior environment of time‐lapse culture provides a more pronounced benefit during extended culture. This suggests that the cumulative impact of atmospheric stability and reduced handling “compensates” for baseline patient risks, especially in patients undergoing multiple transfer cycles.

Previous studies, such as those summarized in the meta‐analysis by Bhide et al. [18], have often been limited by small sample sizes, single‐center designs, or insufficient statistical power to detect meaningful differences in clinical pregnancy or live birth rates. In contrast, our study utilized a prospective sibling‐oocyte allocation design, randomizing oocytes from the same retrieval cycle. This within‐patient randomization effectively minimizes inter‐patient confounding factors—such as maternal age, ovarian reserve, and ovarian stimulation protocols—offering a more precise and controlled comparison of the two incubation systems. By directly comparing sibling embryos, our design significantly strengthens the internal validity of the observed differences in embryo quality and clinical outcomes, directly addressing a primary limitation of previous trials.

We acknowledge that the multicenter nature of this study introduced variability in laboratory practices, including the use of both humidified (Geri) and non‐humidified (EmbryoScope) time‐lapse systems, as well as differences in culture media and mineral oil protocols across participating clinics. While such heterogeneity is inherent in multicenter designs, our within‐patient sibling‐oocyte allocation effectively neutralized these variables, as both study and control cohorts were subjected to identical center‐specific protocols. Therefore, the observed improvements in embryological and clinical outcomes likely reflect the inherent advantages of the time‐lapse environment—such as atmospheric stability and reduced handling—rather than center‐specific protocols.

## 5 Study Limitations

Despite its prospective design, this study has limitations. The multicenter approach, while increasing generalizability, introduces slight inter‐center variability in laboratory environments and embryo grading. Additionally, our cohort was restricted to first‐time retrieval cycles; thus, these results may not apply to patients with repeated implantation failure or refractory infertility [16]. Furthermore, the sibling‐oocyte model effectively controlled for patient‐specific variables; the sample size for fresh embryo transfers was relatively small, which may have limited the statistical power to detect significance in that subgroup. Finally, while our study was sufficiently powered for our primary embryological endpoint (good‐quality blastocyst rate), the sample size for fresh embryo transfers was relatively small, which limited our ability to draw definitive conclusions regarding secondary clinical outcomes, such as clinical pregnancy rates, in that specific subgroup, and further research is required to evaluate cumulative live birth rates and long‐term neonatal health.

## 6 Conclusion

In conclusion, this prospective multicenter study provides robust evidence that time‐lapse culture systems offer a superior environment for embryo development compared to conventional culture. By utilizing a sibling‐oocyte design, we successfully isolated the effect of the culture environment from patient‐specific confounding factors, demonstrating that time‐lapse monitoring significantly improves the proportion of good‐quality embryos on Day 2 and good‐quality blastocysts.

Our findings contribute to the advancement of reproductive medicine by validating the diagnostic utility of time‐lapse monitoring in detecting early pronuclear fading and abnormal cleavage patterns—critical events that are often undetected in standard protocols. While clinical pregnancy rates showed a higher tendency in the time‐lapse group, we acknowledge that these secondary outcomes were exploratory and that the current study was not powered to evaluate live birth or cumulative outcomes. Consistent with recent large‐scale evidence, such as the TILT trial (2024), our results suggest that while stable laboratory environments significantly optimize embryological quality and selection, further research is required to determine the definitive impact on cumulative live birth rates and long‐term neonatal follow‐up. These findings support the integration of time‐lapse technology as a powerful tool for optimizing laboratory performance and moving toward more data‐driven clinical practice.

## Funding

This research was supported by AMED under Grant number JP22gk0110056.

## Disclosure

The authors have nothing to report.

## Ethics Statement

This study was supported by the Japan Agency for Medical Research and Development (AMED) under Grant No. JP22gk0110056 and was approved by the JISART Ethics Committee (Registration No. 2021‐06). All procedures involving human participants were performed in accordance with the ethical standards of the relevant institutional and national research committee and the 1964 Declaration of Helsinki and its later amendments.

## Consent

Informed consent was obtained from all patients.

## Conflicts of Interest

The authors declare no conflicts of interest.

## Data Availability

The data that support the findings of this study are available from the corresponding author, Y.M., upon reasonable request. To protect participant privacy, the raw clinical data are not publicly available.
